# GIS measured environmental correlates of active school transport: A systematic review of 14 studies

**DOI:** 10.1186/1479-5868-8-39

**Published:** 2011-05-06

**Authors:** Bonny Yee-Man Wong, Guy Faulkner, Ron Buliung

**Affiliations:** 1 Faculty of Physical Education & Health, University of Toronto; 2 Department of Geography, University of Toronto Mississauga

## Abstract

**Background:**

Emerging frameworks to examine active school transportation (AST) commonly emphasize the built environment (BE) as having an influence on travel mode decisions. Objective measures of BE attributes have been recommended for advancing knowledge about the influence of the BE on school travel mode choice. An updated systematic review on the relationships between GIS-measured BE attributes and AST is required to inform future research in this area. The objectives of this review are: i) to examine and summarize the relationships between objectively measured BE features and AST in children and adolescents and ii) to critically discuss GIS methodologies used in this context.

**Methods:**

Six electronic databases, and websites were systematically searched, and reference lists were searched and screened to identify studies examining AST in students aged five to 18 and reporting GIS as an environmental measurement tool. Fourteen cross-sectional studies were identified. The analyses were classified in terms of density, diversity, and design and further differentiated by the measures used or environmental condition examined.

**Results:**

Only distance was consistently found to be negatively associated with AST. Consistent findings of positive or negative associations were not found for land use mix, residential density, and intersection density. Potential modifiers of any relationship between these attributes and AST included age, school travel mode, route direction (e.g., to/from school), and trip-end (home or school). Methodological limitations included inconsistencies in geocoding, selection of study sites, buffer methods and the shape of zones (Modifiable Areal Unit Problem [MAUP]), the quality of road and pedestrian infrastructure data, and school route estimation.

**Conclusions:**

The inconsistent use of spatial concepts limits the ability to draw conclusions about the relationship between objectively measured environmental attributes and AST. Future research should explore standardizing buffer size, assess the quality of street network datasets and, if necessary, customize existing datasets, and explore further attributes linked to safety.

## Background

In the context of increasing prevalence of obesity and overweight in children and youth [1], the consideration of active school transport (AST) as an important and utilitarian source of physical activity is of interest. Children who walk to school are more physically active than children who are driven [2]. However, there has been a consistent decline in the use of active modes (i.e., walking, biking) to and from school observed in Western nations [3]. For example, in the Greater Toronto Area, Canada's largest city-region, walking mode share for trips to school declined between 1986 and 2001 (53%- 42% for 11-13 year olds, 39%-31% for 14-15 year olds) [4] while car trips have increased. The immersion of children into the culture of automobility, through parental/caregiver decisions regarding mode choice for daily activities, could establish both short and long-term (through adolescence and into adulthood) expectations regarding mobility that place the automobile at the centre of everyday life. As lifelong patterns of physical activity are established in childhood [5], encouraging and enabling active transportation for daily activities at a young age may be beneficial over the long-term in terms of meeting urban planning and public health goals oriented toward the production of active, healthy, and sustainable lifestyles.

A wide range of correlates of active travel to and from school have been studied including demographic, individual, family, school, social and physical environmental factors [3]. McMillan [6] developed one conceptual framework to examine children's school transportation behaviour that incorporates these commonly examined factors. In her framework, parents are assumed to make the ultimate decision about whether their child can walk to school. This decision is indirectly related to 'urban form'. That is, aspects of urban form are processed by parents and their perceptions, beliefs and attitudes (e.g., regarding traffic or neighbourhood safety) mediate their decisions about their child's school travel. Socio-demographic variables, such as socioeconomic status, may also interact with these perceptions to influence parents' final decisions about school transport mode.

Given the conceptualized importance of the environment in the context of AST in McMillan's [6] and other frameworks (e.g., Panter et al. [7]), objective measurement of the separate but related dimensions of urban form - i.e., the organization and physical form of land use and transportation (systems and services) is crucial to moving from a conceptual to an empirical understanding of school travel behaviour. Existing studies on physical activity, however, have largely relied on self-report measures of the environment [8].This may be appropriate if it is how the elements of the environment are perceived by parents that is critical to the behavioural outcome [3]. However, physically active participants may be more aware of how their neighbourhood facilitates physical activity than inactive ones (e.g., walkers may know better the location of streets with sidewalks than those who do not walk as often). Therefore, active and inactive research participants located within the same neighbourhood may indeed have very different perceptions about the environment they live in. Hence, measuring aspects of the built environment subjectively (e.g., through self-report) may not accurately assess the effect of the actual BE on AST. Accordingly, objective measurement of the built environment, informed by an understanding of how the built environment is constructed (with regard to policy and planning), derived from Geographic Information Systems (GIS) - enabled analyses of digital representations/models of the land use and transportation elements of the built environment, is a necessary complement to self-report and/or qualitative assessment.

The built environment may influence travel demand across three general dimensions--density, diversity, and design, the so-called 3Ds [9], and these qualities may be measured around the home, school, or routes to and from school [10]. Regarding density, compact neighbourhoods may encourage non-motorised travel and reduce single occupant vehicle (SOV) travel by bringing origins and destinations closer together. Moreover, compact neighbourhoods could increase non-motorised travel in other ways such as having greater land use mix, less parking, and improved transit level of service. Distance can be considered as an operational measure of the concept of 'density.' For example, a higher density of schools within a city should produce shorter school trips, on average, than a more sparsely populated geographical distribution of schools. Similarly, land use diversity, characterized by having a mix of destinations potentially makes it more convenient to develop trip chains across a set of activities using active modes such as walking or biking. Design features, including the street pattern (e.g., gridded street patterns have greater connectivity), and pedestrian and cyclist infrastructure, may increase the accessibility of different destinations by non-motorized travel. In addition, design features such as streets with shaded trees can represent an aesthetic that may be appealing to those considering the use of non-motorized modes for short trips.

This 3Ds framework was originally applied to the context of adult travel behaviour but it can be usefully extended as a framework for exploring children's school transport and organizing existing literature on the subject. Several systematic reviews [3,7,8,11] have examined the impact of the built environment on children's AST or active transport. For example, short distances [8], having walking or cycling paths [7,8], few hills [11], and route directness [11] have been found to be positively associated with AST. These findings are primarily based on self-report. Pont and colleagues' recent systematic review [8] only included 4 studies which measured urban form objectively. Additionally, existing systematic reviews do not explicitly analyse how the built environment was being measured using GIS. Given increasing interest in how the built environment may influence AST, a more detailed systematic review is required to inform research and practice regarding what we currently know about the relationship between objectively measured aspects of the built environment and AST; and to identify methodological implications for researchers interested in examining this relationship.

## Methods

### Searching strategies and databases searched

This review consisted of a search of published literature in the English language. Databases were searched using keywords contained in the title, abstract, MESH headings, or descriptor terms. The search strategies involved three stages: 1) a combination of keywords on active school transport (active school transport, active commuting to (from) school, walking to (from) school, (bi)cycling to (from) school, biking to (from) school, walk to (from) school, cycle to (from) school, mode choice to (from) school, commuting to (from) school, commute to (from) school, child pedestrian, child cyclist, safe route to school, mode to (from) school, travel to (from) school), keywords on the BE (physical environment, urban planning, neighbourhood, BE, walkability, road safety, crime, aesthetic, transportation, traffic, urban design, connectivity, distance, sprawl, socio-economic, trail, open space, greenway) and keywords of GIS (Geographic Information Systems, Geographical Information Systems, GIS); 2) a combination of keywords on active school transport and keywords on the BE; and 3) keywords on active school transport. Databases that were searched included Web of Science (1960 - May 2010), Geobase (1973-May 2010), Scopus (1960-May 2010), Medline (1950 to May week 3 2010), Transport (1960-May 2010) and Sport Discus (1960-May 2010). Previous reviews were also examined. References within identified articles were reviewed for further studies.

### Inclusion/exclusion criteria

Each included study had to have: 1) participants between 5 and 18 years of age (elementary or high school students) as the study sample; 2) GIS as a measurement and/or analysis tool; 3) at least one variable related to the built environment relevant to active school transport as an independent variable; 4) at least one variable related to school transport as a dependent variable; 5) and reported empirical data on the built environment and school transport.

### Systematic review process

**Figure 1:**
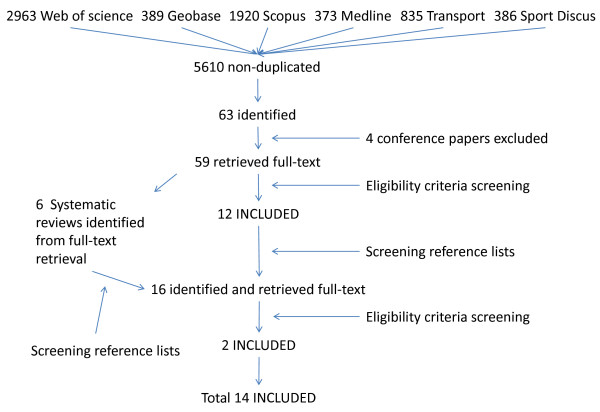
**The flow chart of systematic review process**.

Figure 1 shows the search and retrieval process. The numbers of references searched from each database were 2963 (Web of Science), 389 (Geobase), 1920 (Scopus), 373 (Medline), 835 (Transport), and 386 (Sport Discus). After reviewing each strategy and removing duplicates, 5610 references were found of which 63 were identified following the screening of titles and abstracts. Four were conference papers and not available and hence were excluded. Full texts of 59 publications were retrieved. Six reviews were excluded; their reference lists were reviewed and potential articles were identified. Thirty-six did not measure the built environment with GIS. Another three examined general active transport among children and/or adolescents and were excluded. Two studies were excluded - one was a case study that did not provide statistical data regarding relevant travel mode and built environment relationships [12] and the second study used GIS techniques to estimate the number of school age children in Georgia living within a safe and reasonable walking distance from school [13]. Twelve publications were included at this stage. From reference lists of identified articles and systematic reviews [3,6-8,11,14,15], 16 additional potential publications were identified and their full-texts were retrieved, of which eight did not examine AST, three did not measure the built environment, another two did not measure the built environment with GIS, and one studied general active transport. Ultimately, 14 published studies were included in this review.

## Results

All reviewed studies were cross-sectional (Table 1), most were American [16-22], three were Canadian [23-25], two European [26,27], one Australian [28], and one Taiwanese [29]. Five studies [16,18,19,22,26] included both children and adolescents. Seven studies included children only [20,23-25,27-29] and two included adolescents only [17,21]. For the purpose of description, elements of the built environment examined in these studies have been organized using the 3Ds framework described earlier [9].

**Table 1:** Summary of studies included in this systematic review

	Population				GIS measures			AT measure				
Author (year) (reference code)	Sample size	Age range (years)/Grade range	sex	Country (locality)	Environmental variables	Operational definition of neighbourhood	Geocode	Modes	Data source	Recall period	Classification	% AST
Babey (2009) [21]	3451	12-17	MF	US (California)	Distance to school; urbanisation	Not reported	Not reported	Walk, bike, or skateboard	b	7 days*	Walking or biking or skateboarding to or from school at least once a week	49.3%
Braza (2004) [20]	34 schools (2993 students)	Grade 5	MF	US (California)	Neighbourhood population density; street connectivity	800-meter radial buffer around school	Street address	Walk, bike, car, bus	b	On the day of data collection	Percent of students walking or biking to school	33%
Bringolf-Isler (2008) [26]	1031	6-7; 9-10; 13-14	MF	Switzerland	Distance to school; length of street segment; altitude between home and school; population density	200-meter buffer around the straight-line between participant's home and school	Not reported	Walk, bike/kick scooter/inline skates, car, bus/tram/train or others	a	Usual travel	Usually walking or biking to and from school both in winter and summer	77.8%
Ewing (2004) [19]	709 trips	Grade K-12	MF	US (Florida)	Commercial floor area ratio, street density, average sidewalk width, proportion of street miles with street trees, proportion of street miles with bike lanes or paved shoulders, proportion of street miles with sidewalks	Not reported	Not reported	Travel diary-school trips-walk, bike, bus	b	--	i)Walking and ii) biking	--
Kerr (2006) [16]	259	5-18	MF	US (Seattle)	Neighbourhood and individual walkability index (residential density, mixed land use, intersection density); neighbourhood income	1-km Euclidean and network buffer around home	Street address	Walk, bike, ride in a car or school bus, public transport to and from school	a	Usual travel	Walking or biking to and from school at least once a week	25.1%
Larsen (2009) [23]	810	11-13	MF	Canada (London)	Street trees; intersection density; sidewalk length; land use mix; distance to school; net dwelling density; net residential density; single parenthood; educational attainment; median household	1-mile radial buffer around school and 500-meter radial buffer around home	Postal code	Walk, bike, scooter, skateboard, rollerblade, school bus, city bus, driven in a car	b	Usual travel	Non-motorized vs. motorized i) to school and ii) from school	62% to school and 72% from school
Lin (2010) [29]	330	Grade 1-6	MF	Taiwan (Taipei)	Residential density; employment density; building density; road density; land use; block size; sidewalk width; sidewalk coverage; intersection number along the route to school; vehicle lane width; shade tree density; slope gradient	Not reported	Not reported	Walk, bus, vanpool, motorcycle, car	b	Unknown	Walking i) to school and ii) from school	About 40% walking i) to and ii) from school
Martin (2007) [22]	7433	9-15	MF	US	Geographic regions; urbanisation	Not reported	Not reported	Walk, bike	a	Usual travel	Walking or biking to school at least once a week	47.9%
McDonald (2007) [18]	614	5-18	MF	US (California)	Dwelling units density; employment density; land use mix; residential index; average block size; intersection density; % each way intersections; % on public assistance; % living below poverty line; % female-headed family; % unemployed; % non-white; % foreign born; % owner-occupied housing; % living in same house 1995	800-meter radial buffer around home	Street address	Walk	a,b	2 days	Walking to school	38% for trip less than 1.6 km and 5% greater than 1.6 km
Mitra (2010) [24]	1548 school trips	11-13	MF	Canada (Greater Toronto Area)	Density of school, urbanisation, Employment to population ratio, median household income	Traffic analysis zone (TAZ)	**	Walk	c	1 day	Walking i) to and ii) from school	--
Mitra (2010) [25]	8009 school trips (4009 to school and 4000 from school)	11-13	MF	Canada (Greater Toronto Area)	Distance to school, work/school-trip density, median household income, intersection density, number of street blocks, distance between central business district and home, ratio of sales/service employment to the population, ratio of manufacturing/trade/office/professional employment to the population	400-meter straight-line buffer around home and school	unknown	Walk	c	1 day	Walking i) to and ii) from school	--
Panter (2010) [27]	2012	9-10	MF	United Kingdom (Norfolk)	Road outside child's home; road density; proportion of primary roads; building density; streetlight density; traffic accidents per km; pavement density; effective walkable area; connected node ratio/connectivity; junction density, land-use mix, socioeconomic deprivation; urbanisation (around home) Streetlight density; traffic accidents per km; main/secondary road en route; route directness; percent of route to school within an urban area; land-use mix (along route)	800-meter street network buffer around home and 100-meter buffer around the shortest route to school	Street address	Walk, bike, car, bus, train	b	Usual travel	i) Walking and ii)biking to school	40.0% walking to school and 9.2% biking to school
Schlossberg (2006) [17]	287	Grade 6-8	MF	US (Oregon)	Distance to school; route directness; intersection density; dead-end density; crossing major roads and rail roads	200-meter buffer around the estimated route to school	Street address	Walk, bike, car, carpool, school bus, program van and other	a	Usual travel	i) Walking and ii) biking as primary mode (three days or more a week)	15% to school and 25% from school
Timpero (2006) [28]	912	5-6 and 10-12	MF	Australia (Melbourne)	Distance to school; busy-road barrier; route along busy road; pedestrian route directness; steep incline en route to school; area-level SES	Along the estimated route to school	Street address	walk, bike	a	Usual travel	Never; walking or biking one-four times a week; and five times or more a week	Five times or more a week: 27.2% (5-6 yr); 38.5% (10-12 yr)

^a ^parent-report; ^b ^self-report; ^c ^proxy report from an adult household member;

*Adolescents who were not in school in the past week, but attended school in the past year, were asked about a typical school week.

**The telephone interviews were stratified by Traffic Analysis Zone and these data were aggregated at the level of Traffic Analysis Zone

### Density

Six studies measured residential density as either the number of residential units or the total number of residents divided by the area of residential land [16,18,20,23,26,29] (Table 2). Besides residential density, McDonald et al. [18] measured density using a residential index (housing units divided by total employment and housing units [30]) and employment density. All studies except McDonald (e.g., Traffic Analysis Zone as the spatial unit) [18] and Lin (no information provided) [29] used a Census data block group as the spatial unit of data collection. Census data (or Statistics Canada) [18,23] and land use data from local governmental departments [18] were also typically used (Table 3). However, one study did not report the type of data used [16].

**Table 2:** Existing built environmental measures

Environmental measures	Definition/formula/GIS methods	Scale of measuring the variables	
**Distance**			
Distance to school	Shortest path to school along the circulation system (including roads, trails, and pathways) estimated by GIS/ArcView 3.x extension, Network Analyst V1.0b estimated the distance based on the shortest route possible along road network	--	[17,23,27,28]
	Straight-line distance between home and schools	--	[21,25,26]
	Manhattan distance between school and home	--	[18]
	Not reported		[29]
Distance to Central Business District	Distance between the Toronto Central Business District and Traffic Analysis Zone of a respondent's home	--	[25]
**Density**			
			
Residential/dwelling density	The ratio of residential units to the residential area	Block group/Traffic Analysis Zone	[16,18,23]
	Total number of residents per land area	Block group	[20,26]
	The ratio of total number of residents to the residential area (and commercial use)	Block group	[23,29]***
Residential index	Residential units as a percent of dwelling units and total employment in the traffic analysis zone	Traffic Analysis Zone	[18]
Employment density	Number of employees per land area	Traffic Analysis Zone	[18]
	Employment to population ratio	Traffic Analysis Zone	[24]
	Ratio of sales/service employment to the population	Traffic Analysis Zone	[25]
	Ratio of manufacturing/trade/office/professional employment to the population	Traffic Analysis Zone	[25]
	Number of employees per area of industrial and commercial land	Unknown	[29]
Building density	Area of floor space/buildings per land area	Study area	[27,29]
School density	Number of school per land area	Traffic analysis zone	[24]
Density of school- or work-related trips	Walking density-total work and school related walking trips produced by residents in study area	Traffic Analysis Zone	[25]
Vehicle density	Number of cars and motorcycles per area of roads	Study area	[29]
**Diversity**			
Mixed land use	Land Use Entropy	Block group/Traffic Analysis Zone	[16,18,23,29]***
	Herfindahl-Hirschman index-proportion of each land use squared and summed	Not reported	[27]
Land use intensity for commercial properties	Commercial floor area ratio (FAR) = commercial floor area/commercial land area	Not reported	[19]
Retail floor area ratio	Retail building square footage/retail square footage	Block group	[16]
**Design-connectivity-intersections**			
Intersection density	The ratio of number of intersections 3- to 4-way or 3- to 5-way or not specified) to the land area/street length	Block group/study area	[16-18,20,23,27]
	Number of major road intersection (3 or 4-way) per land area (primary highway, secondary highway and major/arterial roads)	Study area	[25]
	Number of 4-way local street intersections	Study area	[25]
	Intersection number along the route to school	Study area	[29]
Percent of 1,3,4, and 5-way intersections	Percent of 1,3,4, and 5-way intersections with the buffer	Study area	[18]
Connected node ratio	The ratio of number of intersections to number of intersections and cul-de-sacs	Study area	[27]
Cul-de-sac density	The ratio of number of cul-de-sac to land area	Not reported	[17]
**Design-connectivity-route directness**			
Pedestrian route directness	The ratio of the distance to school along the road network to the straight-line distance	--	[17,27,28]
**Design-connectivity-streets**			
Block/road density	Road length (local streets, arterials, and collectors) or number of blocks per land area	Study area	[19]*** [25,27,29]***
Average block size	Not reported	Study area	[18,29]
			
Length of each types of road or all streets	Total length of motorway, main street, and side street (Switzerland) in each study area	Study area	[26]
Proportion of primary road	Length of primary roads per length of all roads		[27]
Vehicle lane width	Average width of vehicle lanes along the route to school	Study area	[29]
**Design-sidewalk and bike lanes**			
Sidewalk/walking tracks length	Total length of sidewalk/walking tracks in the study area	Study area	[23]
Average sidewalk width	Not reported	Not reported	[19]
	Average sidewalk width along the route to school	Study area	[29]
Sidewalk density	Proportion of street miles with sidewalk/pavement	Study area	[19]*** [27]
	Percentage length of sidewalks with widths wider than two metres along the route to school	Study area	[29]
Bike lane density	Proportion of street miles with bike lanes or paved shoulders	Not reported	[19]
**Street spatial design**			
Across a motorway, main street or a side street/across busy road (freeway, highway, arterial, subarterial, collector, and local road)/across major roads, or rail roads	Whether the route to school cross these road	Along the estimated route to school/the straight line between school and home	[17,26,28]
Route along busy/main or secondary road	Whether the route to school along a busy/main or secondary road	Along the estimated route to school	[27,28]
Route along			
Road outside child's home	A major or minor road adjacent to the child's home	--	[27]
Proportion of primary roads	Presence of primary road as part of the route	Along the estimated route to school	[27]
**Walkability index**			
Neighbourhood walkability index	Walkability=[(z net residential density) = (z retail floor area ratio) + (2 × z intersection density) + (z land use mix)]	Cluster of block groups	[16]
Individual walkability index	Walkability=[(z net residential density) = (z retail floor area ratio) + (2 × z intersection density) + (z land use mix)]	Study area	[16]
Effective walkable area	Total neighbourhood area (area that can be reached via the street network within 800 m from the home) divided by the potential walkable area (the area generated using a circular buffer with a radius of 800 m from the home)	Study area	[27]
**Topography and aesthetics**			
Greenery	Proportion of street miles with street trees	Not reported	[19]
	Total number of street trees within 5 m of each road edge	Study area	[23]
	Number of shade trees per the length of route to school	Study area	[29]
Steep incline	Altitude between home and school; detail not reported	Along the straight line between school and home	[26]
	A TIN (triangulated irregular network) file was created from the digital elevation model (data from the State of Victoria). Surface analysis was undertaken along each route to determine the presence of a steep incline along any segment using Surface Tools, version 1.5.	Along the estimate route to school	[28]
	Average slope gradient within residence area of a child	Not reported	[29]
Geographic regions	Northeast, South, Mideast, or West in the US	--	[22]
Urbanisation	Population density>4150 persons per square mile (ppsm) = urban; 1000-4150 ppsm = suburban; <1000 ppsm = rural	Not reported	[21]
	Five levels of urbanisation defined by quintiles of population density and density of the surrounding areas: urban, metro suburban, second city, town, and rural	Not reported	[22]
	Urban, inner-suburban, outer-suburban	Traffic analysis zone	[24]
	Urban or rural based on address of child's home	--	[27]
Proportion of route to school within an urban area	Proportion of route that passes through urban area	Along the estimated route to school	[27]
**Safety**			
Traffic accidents	Number of fatal or serious road traffic accidents divided by total road length	Study area	[27]
Streetlight density	Number of streetlights divided by total road length	Study area	[27]
**Demographic-socio-economic status**			
Area-level socioeconomic status score	Based on the Australian Bureau of Statistics' Index of Relative Socio-Economic Advantage/Disadvantage	Not reported	[28]
Neighbourhood income	Median household income	Block group/Dissemination area	[16,23]* [24,25]
Socioeconomic deprivation	Population weighted scores for index of multiple deprivation	Not reported	[27]
Percent of residents on public assistance		Census tract	[18]**
Percent of household with income below poverty level		Block group/Census tract	[18]**
Percent of residents unemployed		Census tract	[18]**
**Demographic-education**			
Educational attainment at neighbourhood level	Proportion of population over age 25 years with high school diploma	Block group	[23]*
**Demographic-housing**			
Percent of residents living in owner-occupied housing		Block group/Census tract	[18]**
Percent of residents living in the same house as 1995		Census tract	[18]**
Percent of residents living in female headed households		Block group/Census tract	[18]**
**Demographic-ethnicity**			
Percent of persons born abroad		Block group/Census tract	[18]**
Percent of non-white residents		Block group/Census tract	[18]**
**Demographic-single parenthood**			
Single parenthood	Proportion of families headed by single parents	Block group	[23]*

*Canadian equivalent: dissemination area

**Census Tract is a geographic unit comparable to Traffic Analysis Zone which was used to measure other variables.

***Scale of measuring variables is not reported.

**Table 3:** Summary of GIS data sources used

GIS data sources	
**Network data**	
Topographically Integrated Geographic Encoding and Referencing (TIGER)/line street centerline data (US)	[17-20]
City of London Planning Department (Ontario, Canada)	[23]
The State Government of Victoria (Australia)	[28]
Department of Urban Development of Taipei City (Taiwan)	[29]
Bicycle and pedestrian level of service database-County's Geographic Information System (Alachua County, Florida, US)	[19]
DMTI CanMap Route Logistics	[25]
Commercial data: Ordnance Survey Integrated Transport Network	[27]
Aerial images (e.g., Orthophotos, 4-m multispectral satellite imagery (Ikonos), 15-cm resolution [23])	[23] (enhance accuracy)
Field surveys/Audit	[23] (enhance accuracy)
**Demographic/land use**	
Census data (e.g., U.S. Census Bureau/Statistics Canada/Australian Bureau of Statistics, Household Registration Office of Wenshan District (Taiwan), etc)	[20,22-26,28,29]
The Metropolitan Transportation Commission and Association of Bay Area Governments (Alameda, California, US) (land use)	[18]
Property appraiser's database (parcel layer in county's GIS) (Alachua County, Florida, US) (land use)	[19]
Commercial data: Ordnance Survey Mastermap Address Layer 2 (land use)	[27]
Derived land cover data (land use)	[27]
House tax database of the Taipei Revenue Service (floor area data)	[29]
Department of Transport of Taipei City (employment, vehicle ownership, and travel speed)	[29]
Police Department of Taipei City (crime)	[29]
Norfolk and Suffolk constabulary (traffic accidents)	[27]
Commercial data (e.g., PRIZM database by Claritas [population density-urbanisation])	[21,22]
**Others**	
Local Authority (streetlight data)	[27]
Commercial data: Ordnance Survey Mastermap Topography (slope data)	[27]
Not reported	[16,26] (network and altitude data not reported) [21]

Three out of nine associations between residential density and AST were positive and the remainder were null [16,18,20,26,29] (Table 4). Two studies found a positive association between residential density and AST in the fifth grade [20] and in children ages 4 to18 years [16]. However, Bringolf-Isler et al. failed to find such an association for youth aged 6-14 years [26]. Larsen et al. [23] reported a significant association between residential density in the home neighbourhood and active commuting back home but not to school in youth aged 11-13 years. Similarly, McDonald [18] only found an association between residential density and active commuting to school for long trips (1.6 km or more) but not for short trips (less than 1.6 km). However, in the same study, associations between a residential index and AST were not found [18]. Macdonald et al. [18] and Lin et al. [29] failed to find an association between employment density and AST. In contrast, Mitra et al. [24] reported an association between employment density and membership in spatial clusters of high AST rates in the morning, they did not find that this relationship held for the afternoon period. Moreover, the type of employment seems to moderate the employment density effect, and the impact of employment density seems to vary over time. In their study of 11 to 13 year olds, Mitra et al. found that the density of manufacturing/trade/office/professional employment had a stronger and negative association with AST for morning trips to school from home, while retail/service employment density had no association with AST [25].

**Table 4:** Summary of relationships between GIS-measured environmental factors and AST

	Individual			Trip			School		
Environmental variables	-ve	+ve	Null	-ve	+ve	Null	-ve	+ve	Null
*Distance*									

Distance to school	[17]^a,b ^[18]^j ^[21,23]^c,d ^[26-28]^a,b,h,i ^[29]^d,m^		[18]^k ^[29]^c^	[25]^c,d^					
Distance to central business district						[25]^c,d,e,f^			

*Density*									

Residential density		[16,18]^k ^[23]^d,e^	[18]^j ^[23]^c,e,f, ^[23]^d,f ^[26,29]^c,d^					[20]	
Residential index			[18]^j,k^						
Employment density			[18]^j,k ^[29]^c,d^	[24]^c^	[25]^c,e,h ^[25]^d,e,f,n^	[24]^d ^[25]^c,f,n ^[25]^c,d,e,f,o^			
Building density		[29]^d,l^	[27]^a,b,e ^[29]^c^						
School density					[24]^d^	[24]^c^			
Density of school- or work-related trips					[25]^c,d,e,f^				
Vehicle density		[29]^d,l^	[29]^c^						

*Diversity*									

Mixed land use		[23]^c,d,f ^[29]^c,l^	[16,18]^j,k ^[23]^c,d,e ^[27]^a,b,e,g ^[29]^d^						
Commercial floor area ratio						[19]^a,b^			

*Design-connectivity-intersections*									

Intersection density		[17]^a ^[29]^c,g,m^	[16,17]^b ^[18]^j,k ^[23]^c,d,e,f ^[27]^a,b,e ^[29]^c,d,e ^[29]^d,g^			[25]^c,d,e,f ^			[20]
Percent of each way intersections			[18]^j,k^						
Connected node ratio	[27]^a,b,e^								
Cul-de-sac density	[17]^a^		[17]^b^						
*Design-connectivity-route directness*									
Route directness	[28]^i ^[27]^a,b^		[17]^a,b ^[28]^h^						

*Design-connectivity-streets*									

Block/road density		[27]^a,b,e^	[29]^c,d^		[25]^d,e^	[19]^a,b ^[25]^c,d,f ^[25]^c,e^			
Average block size	[29]^c,m ^[29]^d,l^		[18]^j,k^						
Length of motorway			[26]						
Length of main street			[26]						
Length of side street			[26]						
Proportion of primary roads			[27]^a,b,e^						
Vehicle lane width			[25]^c,d^						

*Design-Pedestrian-sidewalk and bike lanes*									

Sidewalk length			[23]^c,d,e,f ^						
Sidewalk width			[29]^c,d^			[19]^a,b^			
Sidewalk density		[29]^c,m^	[27]^a,b,e ^[29]^d^		[19]^a^	[19]^b^			
Bike lane density						[19]^a,b^			
Street spatial design									
Rail roads crossing			[17]^a,b,g^						
Motorway crossing			[26]^g^						
Major roads crossing			[17]^a,b,g^						
Main street crossing	[26]^g^								
Side street crossing			[26]^g^						
Busy road crossing	[28]^g,h,i^								
Busy/main road along the route	[27]^a,b^		[28]^h,i^						
Main road outside child's home			[27]^a,b,e^						
Proportion of primary roads			[27]^a,b,g^						

*Walkability index*									

Individual walkability index		[16]							
Neighbourhood walkability index		[16]							
Effective walkable area			[27]^a,b,e^						

*Topography*									

Greenery		[23]^c,e ^	[23]^c,f ^[23]^d,e,f ^[29]^c,d^			[19]^a,b^			
Steep incline	[28]^h ^[29]^d,m^		[26,28]^i ^[29]^c^						
Geographic regions	[22]*								
Urbanisation		[21,22]	[27]^a,b,e,q^		[24]^c,d^				

*Safety*									

Density of traffic accidents			[27]^a,b,q,e^						
Streetlight density			[27]^a,b,e,g^						

*Demographic-socioeconomic factors (income, employment)*									

Area-level SES			[28]^h,i^						
Neighbourhood income	[23]^d,f^		[16] [23]^c,e,f ^[23]^d,e^	[25]^c,d,e,f^	[24]^c,d^				
Socioeconomic deprivation		[27]^a,b,e^							
Percent of residents on public assistance			[18]^j,k^						
Percent of residents living below poverty line			[18]^j,k^						
Percent of residents unemployed			[18]^j,k^						

*Demographic-education*									

Educational attainment at neighbourhood level			[23]^c,d,e,f^						

*Demographic-housing*									

Percent of residents living in owner-occupied housing			[18]^j,k^						
Percent of residents living in the same house since 1995			[18]^j,k^						
Percent of residents living in female headed households			[18]^j,k^						

*Demographic-ethnicity*									

Percent of residents born aboard			[18]^j,k^						
Percent of residents being Black			[18]^j,k^						

*Demographic-parenthood*									

Single parenthood at neighbourhood level			[23]^c,d,e,f^						

*Interactions*									

Neighbourhood walkability × income		[16]							
Neighbourhood walkability × parental concern		[16]							
Distance to school × community	[26]^#^								
Distance to central business district × block density	[25]^c,d,e,##^								

^a ^walk; ^b ^bike; ^c ^to school; ^d ^from school; ^e ^home neighbourhood; ^f ^school neighbourhood; ^g ^en route; ^h ^5-6 years old; ^i ^10-12 years old; ^j ^trip less than 1.6 km; ^k ^trip greater than 1.6 km; ^l ^dependent travel; ^m ^independent travel

^n ^the ratio of manufacturing/trade/office/professional employment to the population; ^o ^the ratio of sales/service employment to the population

*In U.S., adolescents living in South region were less likely to actively commute to school than those in Northeast region.

^#^The strongest relationship between distance between home and school on AST was found in Biel (German-speaking) followed by Biel (French-speaking) and Bern.

^##^Children living in a neighbourhood with smaller blocks and located far from the central business district were less likely to walk than those living in a place with larger blocks and located closer to the central business district.

### Density: Distance

Five studies [17,23,27,28] estimated the distance between school and home using the network analysis capabilities offered within off-the-shelf GIS software. These studies all applied a shortest path algorithm to estimate the travel distance between school and home along a digital street network. Three other studies [21,25,26] estimated school travel distance by measuring the 'straight-line' or Euclidean distance between school and home. One [18] estimated Manhattan distance with the assumption that children walked along a gridded street network. One study did not report how the distance to school was measured [29].

Of all studies reviewed [17,18,21,23,25-29], fifteen out of seventeen reported negative associations between distance to school and i) walking to school [17,18,25,27,29], ii) biking to school [17,27] and iii) walking or biking to school [21,23,26,28]. Two null relationships were reported between distance to school and walking to school [18,29]. Distance was found to be negatively associated with active commuting in Switzerland; however, the strength of such an association varied across different communities [26]. No study identified a positive association. Lin et al. [29] observed an association between distance to school with walking independently back home, but not for walking to school. Moreover, McDonald et al. [18] reported that increasing distance was negatively associated with AST when the trips were short (e.g., less than 1.6 km) and no association was found when the trips were longer than 1.6 km. These findings provide convincing if not conclusive evidence that increasing distance is negatively associated with AST. While it is rather intuitive to conceive of the sort of relationship being tested, it is perhaps more critical, from a policy perspective, to consider broadening our understanding of those processes/forces that are actually responsible for the production of distance.

### Diversity: Land use mix

Four of the reviewed studies included measures of land use mix (diversity). In four studies [16,18,23,29], land-use mix was measured using an entropy index, which quantifies the degree of mixing across land-use categories within a neighbourhood [9]. Some scholars have also used the Herfindahl-Hirschman index[27]. As a measure of land use intensity, Ewing et al., estimated, for each parcel, a commercial floor area ratio (FAR) expressed as the ratio of a parcel's commercial floor area to the parcel's land area dedicated to commercial uses [19]. The majority of these studies specified the source of land-use data: Metropolitan Transportation Commission and Association of Bay Area Government [18], county's (e.g., Alachua County, Florida) [19] and local governmental departments (e.g., City of London Planning Department [23]), and commercial data [27].

Three out of fifteen associations between land use mix and AST were positive [23,29] and the remainder were null [16,18,19,23,27,29]. Larsen et al. [23] reported significant positive associations between land-use mix in the school neighbourhood with AST both to and from school, but no association between land-use mix in the home neighbourhood and AST in youth aged 11-13 years. No association between land-use mix and AST was observed in children aged 4-18 years [16] or 5-18 years [18]. Moreover, Lin et al. report that children living in an area with mixed land use were more likely to actively commute to school dependently (with an adult) but such an association was not found for trips home from school [29].

### Density and Diversity: Walkability Index

One study [16] combined land use, residential density, and connectivity measures to develop a composite walkability index. This index, constructed using the following formula: (z score of net residential density) + (z score retail floor area ratio) + (2 × z score intersection density) + (z score land use mix), normalizes the four components of the walkability index for each block group using a z-score. Students who lived in neighbourhoods with a higher walkability index were more likely to actively commute to school [16]. In high walkability neighbourhoods, children with high socioeconomic status were more likely to actively commute to school than those with low socioeconomic status [16]. Similarly, children from households with low parental concern about safety and barriers in a high walkability neighbourhood were more likely to actively commute to school than their counterparts [16].

### Street Design: Intersection and dead-end densities

Eight studies measured intersection density [9] by dividing number of intersections by the area of spatial units used in the analysis (e.g., 200-meter buffer along the route to school [17], 400-meter [25], 500-meter [23], 800-meter [27] or 1-km buffer around home[16], 1.6-km buffer around school [23] or traffic analysis zones[18]) [16-18,23,25,27] or by dividing the number of intersections by the length of a road segment [20]. Lin et al. examined the number of intersections along the route to school [29]. Intersections were defined in different ways: 3-way or more [16,25], 3- and 4-way [17,23], and undefined [18,20,27,29]. Most studies reported the data used for measuring intersection density (e.g., street centerline/road network data [17,18,20,23,25,29]). Besides intersection density, Schlossberg et al. measured the ratio of number of dead-ends to the area of the 200-m buffer along the shortest route to school [17]. In studies where the relationship between intersection density and AST has been described, null relationships were reported in eighteen of twenty cases [16-18,20,23,25,27,29]; the remaining two cases reported a negative association [17,29]. No association between intersection density and AST was observed for youth aged 9-10 years [27] or 11-13 years [23,25] or for children aged 4-18 years [16] or 5-18 years [18]. However, Schlossberg et al. [17] found a negative association between intersection density and walking, but not biking, to school in grade 6-8 students. Lin et al. [29] found a similar association but only with independent (unescorted) active school transport in the morning.

### Street Design: Use and Route Directness

Pedestrian route directness [31], a connectivity measure defined as ratio of the shortest estimated distance to school along the road network to the straight-line distance, was measured in two studies. Street centerline data [17-20], governmental data [23,28,29] and in one study commercial data (Ordnance Survey Mastermap Transport Network, UK) [27], was input to a GIS for the purpose of estimating numerator data using a shortest path network analysis algorithm.

In these studies [17,27,28], three of the six associations between route directness and AST were negative and the remainder null, meaning that some studies have demonstrated that the requirement for a child to take a relatively indirect route to school typically associates with the use of some form of motorized transportation (typically the private car). Timperio et al. [28] found a significant negative association between route directness and AST in youth aged 10-12 years. The direction of association was the same for children aged 5-6 years but not significant [28]. Panter et al. also reported such negative associations in youth aged 9-10 years. Schlossberg et al. [17] did not report any association for middle school students.

### Street Design: Blocks, Street Length and Availability of Active Infrastructures

Average block size [18,29], length and density of street segments (e.g., main and side streets) in the studied sites (buffer areas of 200 m around the route to school) in the buffer of estimated route to school (straight line between school and home) [26], and street density [19,25,27] in the study area were measured. Only five of fourteen associations between street-related variables and AST were positive and the remainder were null. One study reported associations of street density with walking independently (unescorted) to school and dependently (escorted or with other children) back home [29]. Similarly, Mitra et al [25] reported that children living in an area with higher block density were more likely to walk to school, the relationship did not hold for the trip home from school. Studies that reported associations between street-related variables and AST tend to include younger children and narrower age groups (9-13 years) [25,27,29] whereas studies that failed to report such associations tended to include students from kindergarten to grade 12 [18,19].

Ewing et al. [19] used the Alachua county's bicycle and pedestrian level-of-service database to assess the proportions of street length with bike lanes and sidewalks, average sidewalk width and sidewalk coverage. Similarly, sidewalk completeness (the total length of sidewalk/walking tracks in the study area) was examined in another study [23]. Larsen et al. [23] created a 'circulation system' database by combining the digital maps of road network, trail network, and informal pathways/footpaths, a composite approach that assesses the totality of pedestrian infrastructure (planned and unplanned). No associations were found between densities of bike lanes and sidewalks, average sidewalk width and AST [19,23]. Moreover, Larsen et al. [23] did not find any association between sidewalk completeness and AST in youth aged 11-13 years. In a separate study however, sidewalk coverage was positively associated with school trips by walking but not by biking [19], and with walking independently to school but not back home [29].

### Street Design: Competing Uses and Location

Four studies [17,26-28] examined whether busy roads (e.g., collectors, highways, freeways, rail road, major roads, and arterial) were along or cut across the shortest path estimate of a students' route to school. The findings were mixed. Children aged 5-6 years and youth aged 10-12 years with busy road barriers (freeways, highways, or arterial roads crossing) along their route to school were less likely to walk or cycle to school [28]. Similarly, Bringolf-Isler et al. [26] observed a positive association between main street crossings along a school route and non-active commuting in children in aged 6-14 years. In other studies no associations were found between motorway location, side streets, and railroad tracks crossing the school route and AST [17,26]. Panter et al. [27] reported a negative association between the presence of a main road along the school route and walking or biking to school, while Timperio et al. [28] did not find an association between the presence of a busy road (freeways, highways, or arterial roads) along the school route and AST.

### Street Design: Aesthetics

Three studies measured aesthetics in terms of trees planted along roads [19,23,29]. In one study, trees along roads were counted within 5 meters from road edges in the study area using data from London's Street Tree Inventory [23]. Another study measured the proportion of street miles with street trees using the Alachua County, Florida bicycle and pedestrian level-of-service database [19]. A third approach involved counting the number of trees with shade along the estimated route to school [29].

One out of five associations with AST was positive and the remainder were null [19,23,29]. Larsen et al. [23] found an association between number of street trees within 5 m of the road edge and active commuting to school but not back home for youth aged 11-13 years. Ewing et al. [19] reported no association between proportion of street length with street trees and walking and biking for children and youth ranging from kindergarten to grade 12.

### Street Design: Topography

Three studies examined topography, more specifically, the slope of streets [26,28,29]. Timperio et al.[28] estimated slope associated with school routes by conducting a GIS-based terrain analysis of the study area. A Triangulated Irregular Network (TIN) can be created by fitting a set of non-overlapping triangular facets to a set of irregularly spaced elevation points. This approach creates a vector-GIS representation of terrain from which topographic data can be estimated including slope (rate of change in elevation) and aspect (the direction of maximum gradient). Timperio et al. used the TIN approach to assess the slope of school routes, with a view to determining the presence of a steep incline along any road segment that is part of the set of segments that comprises a student's school travel route [28]. Timperio et al.[28] found a negative association between the steep slope along the route to school and AST in children aged 5-6 years but not in youth aged 10-12 years. Elsewhere, no association between steep slope along the route and AST among youth aged 6-14 years was found [26] while Lin et al.[29] also reported a negative association between steep slope and walking back home independently (unescorted) but not to school in elementary school students. Specific information about how slope was modeled in these two studies was not provided.

## Discussion

There is currently no consistent evidence supporting the association between GIS-measured aspects of the built environment with AST except distance to school. It is important to consider that distance between home and school is produced by interactions between complex social and economic processes that influence home and school locations. For example, and with sufficient capital, people may select themselves into neighbourhoods as an expression of preference for a certain bundle of amenities and services. This process of self-selection could produce residential choices at either end of the sustainability spectrum. Conversely, others may experience a household mobility process where the choice of alternatives is limited to the availability of social-housing at fixed locations across a city, and/or vacancies at the lower end of the rental or owner segments of the housing market. In short, the residential choice process, and housing policy more broadly, has an important role in producing school travel distance.

There was less consistent evidence that land use mix and density and connectivity (intersections) were related to AST, although some studies did find a positive relationship. Other variables such as having a busy road crossing or a busy road located along the route to school; greenery; or composite metrics of neighbourhood walkability have been less frequently assessed, yet in some instances there were either positive, negative or null relationships reported across studies. Does this mean that objective measurement of the built environment is not important to understanding AST? It is premature for such a conclusion at this stage given some of the methodological challenges inherent in this type of research; the possibility that some important features of built environment have not been assessed; the likelihood that the relationship between the built environment and school travel may indeed be different in different cities, regions, and or neighbourhoods; and that the relationships between the built environment and AST may be moderated significantly by a range of other factors such as the age of children and youth, time of day, trip type or chains (e.g., the presence of activities before or after the school trip) or school travel mode.

### Theoretical and Methodological Issues

Subjective environmental measures reflect subjects' perception. Without understanding the process through which subjects experience and interpret their actual environment, the use of objective environmental measurement (e.g., GIS) to assess the effect of BE on AST remains a necessary and complementary methodological approach for understanding this relationship. Despite the importance of GIS measures, there were a number of theoretical and methodological limitations within the current literature including inconsistencies in geocoding, selection of study sites, buffer methods and sizes and the shape of zones (the Modifiable Areal Unit Problem [MAUP]), the quality of road and pedestrian infrastructure data, estimation of the route to school, and inconsistency in applying measures of the built environment. These limitations reflect challenges both in terms of thinking about the spatial science/theory underlying AST research, and the pragmatic/technical understanding of how to apply GIS software.

First, the geocoding issue refers to the accuracy with which a researcher can pinpoint the location of a subject's home location on a digital map of the built environment. This geocoding accuracy issue is moderated by both ethical (i.e., a research ethics board ruling about the use of personal information), and methodological (i.e., data availability) considerations. Moreover, research participants might be reluctant to offer street address locations to researchers. Measures of the built environment attached to a subject's home location could subsequently suffer from measurement error when the subject's home location has not been accurately geocoded. Despite this concern, only one study reported the geocoding method (e.g., postal code geocoding) [23] whereas more commonly, geocoding methods were not reported [16-19,21,22,25-29]. Larsen et al. [23] geocoded students' home based on postal code. However, 25% and 20% of postal code locations were beyond 200 m of the actual street address location; postal code geocoding places subjects within a postal code zone, not at the actual location along the street system [32]. Larsen and colleague's use of a 500 m buffer may have led to substantial error. The issue of compounding error and spatial uncertainty in the location data used in AST research has not been adequately discussed in the literature. Studies that address the sensitivity of experimental results to the geocoding issues are warranted.

Second, a common practice across studies is to generate buffers around home, school, and occasionally route locations, and then to measure the built environment within the buffered objects, using one or more of the approaches described earlier. The expectation is that the presence of enabling infrastructures within buffers, that are often used as a metric for the concept of neighbourhood, will produce active travel outcomes. The primary theoretical concern with regard to buffer analysis involves the specification of buffer methods and size, a process that is subject to the Modifiable Areal Unit Problem (MAUP) [33]. MAUP occurs when the results of data analysis exhibit sensitivity to the geometry (e.g., size, shape) of spatial units (e.g., census zones) used for the reporting of data input to the analysis process. The reference to modifiable areal units reflects the fact that it is quite often the case (particularly with secondary data) that the spatial units under analysis may be arbitrary constructs of a data collection and aggregation process, conducted usually by a third party, with a view to developing spatial units for statistical reporting [33]. The use of buffers in the measurement of built environment characteristics is an example of an analytical process where relatively arbitrary decisions are taken regarding the shape (i.e., AST research usually applies circular buffers, this need not be the case) and size of buffers. The buffer approach is an area-based approach to ascribing built environment qualities to individual cases, because built environment characteristics are estimated within the areal unit of a buffer, these types of measures are likely subject to the MAUP. There has been some discussion of the intersection between MAUP and area-based measures included as independent variables in the multivariate analyses of discrete or continuous outcomes [34,35]. An example to illustrate this issue is presented by Lee's study [36].

MAUP includes two effects: scale and zoning or aggregation effects [33]. The scale effect is the variation in results due to the size of areal units used in the analysis of a given area, which consequently associates with the number of areal units required to exhaustively cover a study area [33]. For example, associations between the built environment and school travel mode may differ between a 400 m or 800 m buffer surrounding the place of residence for the same set of cases. The definition of these units, in this case, the buffers, is arbitrary and modifiable, and hence measures of the built environment derived from buffer analysis, such as counting the number of intersections within a buffer and dividing by the buffer area to generate a measure of intersection density, could change with adjustments to the buffering procedure selected by the researchers. The inconsistency of buffer sizes also makes cross-study comparison difficult. In the reviewed studies, buffer sizes varied from 400 m [25], 800 m [20] or 1.6 km [23] buffers around schools, no buffers [28] or 100 m [27] or 200 m [17,26] buffers around the route to school, 400 m [25], 500 m [23] or 800 m buffers surrounding home [18,27]. Kerr et al. [16] measured the neighbourhood at two-levels: block group and a more proximal one (1 km buffer). One study did not report the spatial units used to measure the built environment characteristics [29]. Moreover, different methods were adopted: Euclidean [17,18,20,23,25,26] and network buffer [16,27]. Based on the original work of Lee and Moudon [10] reflected in the framework described by Panter [7], environments around home, school and en route all impact decision-making on children's AST, and hence it is important to investigate the combined effect of these places and routes on AST. There is, however, an additional statistical issue that requires consideration. In cases where buffers around objects overlap, either because a generous radial distance was selected for buffer creation, or because the buffered objects are simply located very close to one another (e.g., a short school trip), objectively measured built environment data may be highly correlated, an issue that has not been widely discussed [25].

The *zoning *or *aggregation effect *refers to how analytical results may vary when scale is maintained (e.g., the number of units is consistent) but the partitioning (geometry) of the units is adjusted. For example, estimated AST rates for a set of ten traffic zones may be very different when the boundaries for each zone are adjusted to include and/or exclude individual cases. The zoning effect is a geographical problem that has remained hidden from view in AST studies that have made use of exogenously and arbitrarily constructed systems of census or traffic zones for the reporting of either built environment data or school travel mode share. Currently, there is no universal solution for MAUP; and arguably, it is quite useful to conduct policy-based analysis using systems of zones embedded within the discourse surrounding the particular policy issue (i.e., if a school board is evaluating transport policy within a school district, then reporting empirical results at that scale is likely to be the most policy relevant course of action). However, using the most spatially disaggregated data, and demonstrating the sensitivity of the results to both scale and zoning effects increases confidence that the results at the most disaggregated level have some meaning and are not simply the artefact of the ways in which data are being arranged [37]. Surprisingly there has been no theoretical or empirical engagement with this issue in the AST literature.

Third, missing pedestrian data [38] and inaccuracy and incompleteness of street network data [39] could lead to inaccurate measurement of connectivity and the estimation of routes actually used by pedestrians. In this review, using street network data to measure connectivity (e.g., route directness) and pedestrian infrastructures was common [17,28]. However, street network data did not typically include pedestrian options other than streets (e.g., paths or trails). Incomplete pedestrian data (e.g., street centerline network data likely does not include paths or trails that pedestrians could walk) creates uncertainty with respect to measuring connectivity and therefore calls into question what we actually know about the empirical relationship between connectivity and AST. In addition, one study [39] reported variations in the quality of road network datasets in terms of completeness, accuracy, and currency, demonstrating the importance of examining the quality of available road datasets. If necessary, they suggest customizing the data (e.g., by updating with aerial photographs and tax parcels and fieldwork with GPS [39]).

Fourth, the actual route to/from school taken by study subjects has not been assessed in the reviewed studies. Five studies examined the impact of the BE along students' route to school on AST [17,26-29]; however, they used a network shortest path route from a GIS to estimate the route to school. The estimated route, based on the assumption of minimizing the generalized cost of the trip measured in terms of total time or network distance, may not be the actual route taken by the subjects. It is common that children (encouraged by caregivers or not) may look to organize themselves with other children on the way to/from school, this organizational process during the trip may indeed require the use of longer routes than predicted by a shortest path algorithm. Duncan et al.[40] found that the route measured by GPS or GIS were comparable in terms of distance; however, the quality and/or spatial structure of the route was significantly different [40]. That is, the actual routes tended to be less busy, and the data suggest differences in the intersections, turns, and segments traversed. The route distance to school estimated by GIS may be a good proxy but the geography of the shortest route data may not match with the actual route taken by research participants. As a result, measures of built environment characteristics taken within a buffer around a shortest route may not accurately reflect the built environment characteristics that a subject actually experiences. Of course, the shortest path does control for the street architecture, something that is not controlled for at all when applying the Euclidean or Manhattan metrics. Interestingly then, none of the reviewed studies reported the validity of the application of the shortest path approach (e.g., mapping activities or the use of GPS could be used to validate such an approach).

Fifth, there is inconsistency in how GIS measures of the built environment are applied. An example is land use measures. Land use measures used in included studies are entropy [16,18,23,29], Herfindahl-Hirschman Index [27], and a commercial floor area ratio (FAR) [19]. The typical entropy approach to measuring land use mix can be expressed using the following formula:

where *p_j _*is the proportion of land in use *j *and *k *represents the total number of land uses (single family, multi-family, retail/service, and manufacturing/trade/other). The result is an index ranging between zero (single use) and one (mixed land use) [9]. The Herfindahl-Hirschman index was calculated by:

where *K *is the number of land use types, and *P_i _*is the percentage of each land use type (e.g., farmland, woodland, grassland, uncultivated land, other urban, beach, marshland, sea, small settlement, private gardens, parks, residential, commercial, multiple-use buildings, other buildings, unclassified buildings, and roads) within the study area [27]. As a measure of land use intensity, a commercial floor area ratio (FAR) was expressed as the ratio of a parcel's commercial floor area to the parcel's land area dedicated to commercial uses [19].

In summary, inaccurate geocoding, inconsistent selection of study sites, buffer methods and sizes, poor quality of road and pedestrian infrastructure data, inaccurate estimation of the route to school, and inconsistent application of measures of BE attributes were the main methodological challenges identified in the current literature. These challenges are not easily addressed. Standardising the operational definition of neighbourhood and examining the impact of various buffer size on the relationships between BE and AST should be considered in future studies. Future studies should attempt to customise road and pedestrian infrastructure data, if necessary and feasible, to improve quality. Measuring students' route to school may be performed more accurately with GPS or by asking the subjects (or guardians) to map their route to school and then digitising to GIS [41] although these approaches are not without limitations (e.g., lack of signal or signal dropout [40] of GPS or misreading the map by the subjects, not to mention the resource-intensive nature of compiling such data).

### Are we measuring the right thing?

According to frameworks by McMillan [6] and Panter [7], the built environment may influence parents' or/and children's environmental perception which in turn may influence school transport behaviours. It is possible that features of the built environment currently assessed by GIS are not relevant to parents. It is important to compare whether the perceived environment is more explanatory than the actual environment in predicting AST. One study examined the effect of parent's and children's perceived environment and objectively-measured built environment with AST [28]. Similarly, another study [16] examined whether parental concern and perceived environment explained the association between the built environment and AST. In a 'combined' model, perceptions regarding the presence of walk and bike facilities, and the perceived availability of stores within a 20 minute walk remained positively associated with AST, while an objective walkability index did not associate with AST. This may suggest that perceptions of environment, which are incidentally partially a response to the "built" environment presented to the subject, are more powerful predictors than objective measures. However, the self-report and objective measures of built environment did not measure the same thing (e.g., self-report of having no streetlights and objective measure of the route along a busy road in Timperio's study [28], and objective walkability index [42] and self-report of the presence of walk and bike facilities in Kerr's study [16]). It is difficult to compare the independent effects on AST. More specifically, "people's perceptions may, in fact, motivate their behaviour more than the true nature of the situation" [15]. Despite this, both parental perception of the environment and the actual environment may have independent effects on AST decisions. Therefore, in future studies, it is important to ideally combine objective measurement of the environment with the perceptions of parents and children through self-report. To this end, there is likely a need to work on advancing our understanding of how to describe the built environment in non-technical terms to study subjects, using concepts and language that can be translated again back into the frames of reference applied to the planning and engineering of neighbourhoods and cities.

It is also important to identify features of the built environment that have not yet been examined but may be important. For example, perceived safety-related variables may be critical (e.g., reaction to the presence/absence of pedestrian and cycling infrastructure [7,8], controlled intersection (e.g., crossing or green lights at intersections) [11,28], and parental and adolescent safety concerns [7]) may have an effect on AST. While safety is acknowledged as an important factor in the general AST literature [3], few studies have looked to examine this construct using GIS derived variables. Only two reviewed studies examined busy roads crossing [17,26]/along [27,28] the route to school as indicators of road safety. Only one study examined the effect of density of traffic accidents on walking or biking to school; however, no associations were found [27]. Different road safety indicators should be examined in future research. Some road safety indicators in studies on youth's general active transportation or school neighbourhood walkability such as roads with speed humps, chicanes and sections of intentionally narrowed road, and traffic lights [43], average annual daily traffic volume, and percent of high-speed streets [44] could be applicable to the context of AST.

Another safety-related variable, self-reported presence of a controlled intersection (e.g., intersections with crossing and green lights), which was found to be associated with AST [28], has not been examined objectively using GIS. No association was found between GIS-measured intersection density and AST in the reviewed studies. This finding may be attributed to all streets being included (e.g., busy arterials and local streets). However, crossing busy roads (intersections with busy roads) may be considered as a traffic danger and may reduce the likelihood of actively commuting to school if children have to use these intersections. Measuring connectivity without major roads [45] may be more suitable in AST research. For child pedestrians, routes to school using minor roads with less traffic volume and lower speed limits tend to be chosen [40].

Consideration should be given to the hierarchical construction of road networks and the relationship between the different levels of the hierarchy (e.g., large arterials with fast moving traffic, to local streets with sidewalks), and AST outcomes (see for example [46]). Two studies examined the effect of intersection density without major streets [27] or with local streets only [25] on AST; however, no association was found. There is more work to be done on the links between roadway hierarchy and AST. Measuring connectivity, and potentially route directness, without major roads may reflect more appropriately the walkable options that parents would allow their children to take. Besides road safety, parental concerns of safety includes personal safety as well [7] although none of the reviewed articles examined personal safety. The spatial analysis, using GIS, of crime data [44] could be an option in this context.

The frameworks by Panter et al. [7] and Lee and Moudon [10] highlighted the importance of examining the effects of the two trip-ends (home and school) and their surrounding neighbourhoods and the route to school on AST. Only one study in this systematic review examined the neighbourhood surrounding home, school characteristics, and the environment surrounding the route to school [27]. The high ratio of intersections to intersections and dead-ends, low road density, and high socioeconomic deprivation in the neighbourhood surrounding home were negatively associated with walking to school whereas long distance to school, high route directness, and main route along the route were negatively associated with walking to school. No associations between school characteristics (e.g., presence of a school travel plan, walking bus scheme, cycle path, and pedestrian crossings which were measured by questionnaire completed by head teachers and research audit) and walking to school were observed; however, the neighbourhood surrounding schools was not assessed. Mitra et al. [21] reported distinct effects of the neighbourhood surrounding school and home on school transport. Findings from these two studies suggest that the built environment surrounding home and school and along the route to school may have distinct effects on school transport. Future studies on school transport should consider the neighbourhood surrounding the two trip-ends (school and home) and along the route to school.

In summary, some of the suggested variables (e.g., controlled intersections-with green lights or crossing) have not been studied or have been only studied once (e.g., busy road crossing the route to school). Future studies should consider exploring safety further using objective measurement (e.g., intersections with green light, streets with crossing, speed humps, overhead street lights, average annual daily traffic volume, crash rates, crime rates); confirming the spatial design of street networks (e.g., busy roads crossing the buffers or nearest distance to a busy road); modifying connectivity measures (e.g., comparing intersection indicators with and without removing the busy roads and re-examining the connectivity with improved pedestrian and cyclist infrastructures including pathways, trails, bike lanes, etc.); examining environmental features along respondents' route to schools, and assessing neighbourhoods surrounding both school and home and along the route to school.

### Potential modifiers

In addition to the theoretical and methodological challenges in applying GIS to the examination of the built environment and school transport, our findings also suggested that there may be several potential modifiers of any relationship including: age, direction of the route, and travel mode. First, there is the need for researchers to make a distinction between the trip to school, and the trip from school [4]. Larsen et al. [23] observed associations of residential density and street trees along the road in the home but not school neighbourhood with AST. However, different buffer sizes applied to school (1.6 km) and home (500 m) make it difficult to interpret the findings as to whether the built environment has different effects on different school trips or whether the findings are attributed to differences in buffer size (i.e., MAUP effects). However, the finding does highlight the possibility that the influence of the built environment varies temporally. For example, Mitra et al. found an association of density of schools with clusters of high AST only in the morning and an association of employee density with clusters of high AST only in the afternoon [24]. In another study, Mitra et al. [25] found that more built environment variables were associated with walking in the morning than in the afternoon. The temporal variation in associations between the built environment and active school transport may be explained by parental/caregiver schedules (e.g., work) and resource availability. For example, parents may be available to drop their children off along their way to work; however, these working parents may not be available to pick their children up after school [47]. This could lead to the mode shift from passive in the morning to active in the afternoon. Besides temporal variation, the association between built environment trip and school transport may vary between trip-ends (e.g., school and home neighbourhoods). The built environment near the location of residence has been shown to be more strongly correlated with mode choice than the built environment around the school [25].

Second, half of the reviewed studies combined children and adolescents in analyses [16,18,19,26]. The association between number of street trees within 5 m of road edge and active commuting to school was observed in youth aged 11-13 years [23]. In contrast, Ewing et al. [19] failed to find such an association for kindergarten to grade 12 students. A significant negative association between the steep slope along the route to school and AST was found in children aged 5-6 years but not aged 10-12 years [28]. No association between steep slope along the route and AST was observed among youth aged 6-14 years [26]. This suggests that the effect of built environmental features on AST may vary not only across age but within narrow age groups (e.g., early elementary students, late elementary students, middle school students, and high school students [able to drive]).

Third, the effect of environmental features on biking and walking are likely to be different. These modes require different levels of investment by private and public stakeholders in equipment and infrastructure, they typically operate using different parts of the road system, require different skill sets, and the development of the necessary infrastructure may be operationally embedded within very different and sometimes highly contested planning processes. Ewing et al. [19] found a positive association between sidewalk density and walking to school but not biking to school. Similarly, Schlossberg et al. [17] reported a positive association between intersection density and walking to school but not biking to school. However, it is uncommon to examine biking and walking separately (only two studies in this review did so [17,19]). For example, the effect of steep slope on biking and walking to school may be different. In one of the reviewed studies, steep slope was found not to be associated with overall AST [26]. In Canada, few students cycle to school [4] and hence it may not be possible to examine this interaction in the Canadian context, at the population level. However, in European countries where cycling is more prevalent, researchers should consider the interaction between active travel modes and the built environment. In general, the evidence that is available, coupled with our understanding of the planning and practice of walking and biking raises valuable questions about the efficacy of modelling walking and biking together as a single mode category.

In summary, potential modifiers include age (e.g., middle school students and high school students), school travel mode (e.g., walking and biking), direction of route (e.g., to school and from school), and trip-ends (home and school). Important associations between the built environment and school travel mode may be attenuated if these modifying variables are not controlled for. Hence, it is important for future studies to examine their potential interactions with the built environment. If interactions are found, they should be analysed separately.

## Conclusions

The application of GIS to the study of AST is relatively new, but informed by more than a decade of work where GIS has been applied in studies of adult travel behaviour and urban form. Aside from distance, commonly assessed features of the built environment are not consistently related to AST although we acknowledge that some relevant studies may have been excluded if they were unpublished or not in the English language. Numerous methodological challenges exist including inconsistencies in geocoding, selection of study sites, buffer methods, and sizes, incomplete road and pedestrian and cyclist infrastructure data, and inaccurate estimation of the route to school. The use of different definitions applied to similar environment features (e.g., number of intersections per unit of area vs. percentage of particular type of intersection [for example, 3-way] or street density vs. total street length) makes it difficult to compare studies. To facilitate international comparison, including generic measures such as the walkability index, is recommended in future research. Future research should also attempt to a) standardize buffer methods and explore the impact of different buffer sizes on the associations between the built environment and AST, including both school and home neighbourhoods; b) customize the road and pedestrian and cyclist infrastructure data to improve quality; c) measure the actual route to/from school with more accurate measurement (e.g., GPS or map drawing); d) include both objective and subjective measures of the built environment; and e) consider the potential interactions of age, trip direction, and travel mode with the built environment measurement. While such rigor may not always be possible given the accompanying resource demands, future studies certainly need to report greater methodological detail to facilitate replication.

## Competing interests

The authors declare that they have no competing interests.

## Authors' contributions

BYW conducted the literature search, data extraction, literature screen, and data analysis and wrote the initial draft of manuscript. GF and RB participated in literature screening, analysis, providing methodological input, and in the writing of the manuscript. All authors read and approved the final manuscript.

## Authors' information

BYW is a doctoral candidate in the Faculty of Physical Education and Health, University of Toronto and is a *CIHR/HSFC Fellow in Population Intervention for Chronic Disease Prevention*. GF is an Associate Professor in the Faculty of Physical Education and Health, University of Toronto. RB is an Assistant Professor in the Department of Geography, University of Toronto.
